# Emerging Mechanisms and Targeted Therapy of Ferroptosis in Neurological Diseases and Neuro-oncology

**DOI:** 10.7150/ijbs.72251

**Published:** 2022-06-27

**Authors:** Yajie Wang, Bufu Tang, Jinyu Zhu, Junchao Yu, Junguo Hui, Shuiwei Xia, Jiansong Ji

**Affiliations:** 1 Key Laboratory of Imaging Diagnosis and Minimally Invasive Intervention Research, the Fifth Affiliated Hospital of Wenzhou Medical University, Lishui, 323000, People's Republic of China.; 2 School of medicine, Lishui University, Lishui, 323000, People's Republic of China.; 3 Department of Radiology, School of Medicine, Lishui Hospital of Zhejiang University, Hangzhou 310016, People's Republic of China.

**Keywords:** ferroptosis, iron metabolism, GSH, GPX4, lipid peroxidation, neurodegenerative diseases

## Abstract

Ferroptosis is a novel type of cell death characterized by iron-dependent lipid peroxidation that involves a variety of biological processes, such as iron metabolism, lipid metabolism, and oxidative stress. A growing body of research suggests that ferroptosis is associated with cancer and neurodegenerative diseases, such as glioblastoma, Alzheimer's disease, Parkinson's disease, and stroke. Building on these findings, we can selectively induce ferroptosis for the treatment of certain cancers, or we can treat neurodegenerative diseases by inhibiting ferroptosis. This review summarizes the relevant advances in ferroptosis, the regulatory mechanisms of ferroptosis, the participation of ferroptosis in brain tumors and neurodegenerative diseases, and the corresponding drug therapies to provide new potential targets for its treatment.

## Ferroptosis and its Mechanism

Cell death is essential for normal human development, maintaining homeostasis, and preventing overproliferation diseases, such as cancer 1. In the past, almost all types of regulatory cell death in mammalian cells were thought to be caused by the activation of apoptosis 2, but it has recently been found that several regulated nonapoptotic cell death pathways are activated in specific disease states. Ferroptosis is a newly discovered programmed type of regulated cell death that is driven by the accumulation of lipid-based reactive oxygen species (ROS) closely related to the oxidative stress response and cystine metabolism as a regulatory form of nonapoptotic cell death. Mitochondrial shrinkage and lipid peroxide accumulation occur during ferroptosis, unlike in traditional apoptosis, necrosis and autophagy 3, but cell shrinkage, chromatin agglutination and other phenomena do not occur during ferroptosis. When the balance of the intracellular oxidation-reduction system is disrupted, phospholipid molecules containing long chains of unsaturated fatty acids on the cell membrane or organelle membrane are oxidized and destroyed by lipid peroxides 4, resulting in cell membrane rupture and cell death. Since the lipid peroxide production process depends on the presence of iron ions in the cell, this type of cell death is referred to as ferroptosis.

### Mechanism of cellular ferroptosis

Ferroptosis is a programmed pattern of cellular death that is closely related to intracellular amino acids, lipids, and iron metabolism. In addition, other metabolic pathways and related factors can also affect the sensitivity of cells to ferroptosis. Ferroptosis is triggered by the inactivation of the cellular glutathione-dependent antioxidant defense system, leading to the accumulation of lipid ROS 5. The essence of cellular ferroptosis is intracellular lipid oxide metabolism disorders, abnormal metabolism under the catalytic action of iron ions, a weakened antioxidant capacity of cells, and the accumulation of lipid reactive oxygen species, which results in an imbalance in intracellular redox and induces cell death.

#### Ferroptosis and amino acid metabolism

Cystine/Glutamic acid metabolism plays an important role in ferroptosis 6. The Xc-system is a reverse transporter on the cell membrane consisting of light-chain SLC7A11 and heavy-chain SLC3A2 connected by disulfide bonds, transporting glutamate outward and cystine inwards in a 1:1 ratio 7. Cystine is transported to cells and reduced to cysteine, which binds to glutamic acid and glycine to produce glutathione (GSH). Glutathione is an important antioxidant that protects cells from oxidative damage and is the substrate for the lipid repair function of glutathione peroxidase 4 (GPX4). GPX4 is a key enzyme in the antioxidant system, with GSH as the reducing agent, catalyzing the conversion of the peroxide bond into a hydroxyl group, and converting the peroxide into a lipotoid alcohol, causing it to lose its oxidative activity, thereby inhibiting ferroptosis. Erastin and Ras selective lethal small molecule 3 (RSL3) are ferroptosis activators 8. Studies have shown that Erastin inhibits its activity by binding to SLC7A11, affecting cystine transport, and reducing GSH synthesis, resulting in cells failing to remove lipid peroxides in time. This ultimately causes damage to cell membranes, which can trigger ferroptosis 9. RSL3 inactivates GPX4 by covalently binding to GPX4 and disrupting the cellular redox equilibrium, leading to increased lipid peroxidation and inducing ferroptosis 5. In addition, transcription factors can initiate the expression of SLC7A11 to regulate ferroptosis, with the nuclear transcription factor NEF2 as a positive regulator to upregulate the expression of SLC7A11 and tumor suppressor genes TP53, BAP1, BECN1 as negative regulators of SLC7A11 10-12. Therefore, finding drugs that target the key molecule in the ferroptosis-GSH metabolism process is of great significance for the study of ferroptosis.

#### The role of lipid peroxidation in ferroptosis

Reactive oxygen species (ROS) are byproducts of cellular metabolism. In normal metabolic processes, ROS are involved in maintaining stability and in cell signaling. Under pathological conditions, excessive accumulation of intracellular ROS can lead to cell death 13. Studies have shown that lipid peroxides are a key mediator of many pathological conditions, including inflammation, cancer, and neurodegenerative diseases 14. Lipid peroxidation is triggered by OH• to form lipid radicals and lipid peroxyl radicals, which react with polyunsaturated fatty acids (PUFAs) to generate lipid peroxides and eventually cause ferroptosis 15. Iron participates in the accumulation of reactive oxygen species through three pathways 16. ROS react with PUFAs in the lipid membrane 17, 18 to induce lipid peroxidation, thereby triggering intracellular ferroptosis 19, 20. PUFAs contain dyalenyl hydrogen atoms, which easily react with ROS, causing lipid peroxidation and resulting in the death of cellular iron 21. Phosphatidylethanolamines (PEs) containing AA or AA are key phospholipids that induce cellular ferroptosis. Therefore, ferroptosis can be increased by supplementing AA or other PUFAs as well as by inhibiting the activity of ACSL4 and LPCAT3 22. The generation of ferroptosis signals requires the formation of PUFA and coenzyme A (CoA) derivatives and their binding to phospholipids, which may be potential targets for the treatment of diseases associated with ferroptosis.

### Iron metabolism and ferroptosis

Iron is an indispensable trace element in the human body and an essential metal for normal cellular function 23. Iron is involved in many physiological processes, such as oxygen transport, cellular respiration, DNA synthesis, and neurotransmitter biosynthesis in the nervous system 24. Iron homeostasis plays an important role in the survival and development of normal cells, as iron deficiency usually leads to anemia, iron accumulation is a hallmark of ferroptosis, and excessive iron can lead to tissue damage and increase the risk of cancer 25. Cellular iron metabolism is controlled by posttranscriptional control of the iron regulatory proteins IRP1 and IRP2 26. IRP1 and IRP2 are iron regulatory proteins that can regulate iron metabolism genes, such as TFRC and FTH1, under normal physiological conditions to maintain the stability of unstable iron pools (LIPs, composed of a small amount of free Fe^2+^).

Iron is present in the form of Fe^2+^ and Fe^3+^, while circulating iron exists in the form of trivalent iron (Fe^3+^) by binding to transferrin (TF). Free Fe^3+^ enters the cell through the membrane protein transferrin receptor 1 (TFR1) and is then stored in the nucleosome, where Fe^3+^ is converted to Fe^2+^ by nucleosome iron reductase-prostate hexame transmembrane epithelial antigen 3 (STEAP3) 27. Fe^2+^ is then transported from the endosome to the cytoplasm via divalent metal transporter 1 (DMT1) 28. In general, Fe^2+^ is stored in the ferritocyte stock protein complex, composed of ferritin heavy chain 1 (FTH1) and ferritin light chain (FTL), to maintain the balance of unstable iron pools and prevent ROS formation 29. Some Fe^2+^ is exported to the extracellular space via the membrane protein ferritin 1 (FPN1) 30, 31. If the uptake, transport, storage and utilization of intracellular iron fails, excess free Fe^2+^ will be deposited in the cell, and the Fenton reaction will be initiated, generating hydroxyl radicals 32 and ROS 33. Then, ROS modifies and interferes with proteins, lipids and DNA, and a series of peroxidation reactions occur with PUFAs on the cell membrane, generating lipid peroxides and resulting in the destruction of the cell membrane structure, ultimately causing cell ferroptosis 34. Iron accumulation is mainly due to barriers, such as membrane iron transporter (FPN) 35, transport ferritin receptor 1 (TfR1), and divalent metal ion transporter 1 (DMT1) 36, resulting in loss of iron transport control. Alternatively, nuclear receptor coactivator 4 (NCOA4)-mediated degradation of the ferritin phagocytosis pathway can occur, resulting in increased iron storage, followed by Fenton reaction/mitochondrial damage/LOX function, leading to increased iron concentration in the active iron pool (LIP) 37. ROS increases, eventually causing ferroptosis. On the other hand, Fe^2+^ can be used as a cofactor of a variety of metabolic enzymes, enhance the activity of various metabolic enzymes (such as LOXs and PDH1), and promote the production of ROS 25. Therefore, iron metabolism-related factors are potential targets for inducing cellular ferroptosis. However, the specific mechanism by which iron metabolism regulates ferroptosis is not yet clear, and further exploration is needed.

### Ferroptosis due to other causes

Studies have shown that selenium, erythroid 2-related factor 2 (NFE2L2), nicotinamide adenine dinucleotide phosphate (NADPH) and coenzyme Q10 (CoQ10) can also affect ferroptosis sensitivity (Table 1). Selenium is an essential micronutrient for maintaining GPX4 activity, which regulates the abundance and activity of GPX4 by synergistically activating the transcription factors TFAP2c and Sp1, inhibiting ferroptosis to a certain extent to protect neurons 38. NFE2L2 can regulate the expression of related genes by transactivation to limit oxidative damage during ferroptosis, and the NFE2L2 signaling pathway is an important defense mechanism against ferroptosis. The genes regulated by NFE2L2 mainly include those involved in iron metabolism, GSH metabolism, and the anti-ROS process 39. NADPH is involved in the circulation of the GSH-GPX4 antioxidant system, and its heavy consumption will limit the antioxidant function of GSH-GPX4 and induce ferroptosis 40, 41. CoQ10 can be reduced by ferroptosis suppressor protein 1 (FSP1) to prevent lipid oxidation and inhibit ferroptosis 42. Therefore, FSP1 may be an important target for the treatment of related diseases.

### Markers of ferroptosis

Ferroptosis has several special cellular morphological characteristics that differ from other forms of programmed death, mainly manifested in the loss of plasma membrane integrity, cytoplasmic and organelle swelling, and chromatin agglomeration 43. The morphology of mitochondria also changes significantly, including the shrinkage of the mitochondrial volume, increased membrane density, a decreased or absent crest, and ruptured outer membrane. In addition, ferroptosis is accompanied by shedding and aggregation of cells and an increase in intracellular autophagosomes 44. Ultrastructure analysis shows that when ferroptosis occurs,cell membrane rupture and bubbles occur, mitochondria decrease, mitochondria atrophy, the mitochondrial spine decreases or even disappears, and mitochondrial membrane density increases, possibly due to dysfunction of varistor anion channels (VDACs) and alterations in the fluidity of mitochondrial membranes 45. The nucleus is normal but lacks chromatin agglutination, intracellular mitochondria are smaller, the outer membrane of the mitochondria ruptures, and the density of the bilayer membrane increases under electron microscopy. When iron dies, it has the following biological effects: increased ROS, iron ion aggregation, activation of the mitogen-activated protein kinase (MAPK) system 46, 47, inhibition of the Xc-system and an increase in reduced adenine dinucleotide phosphooxidase by reducing cystine uptake, depletion of glutathione, and Xc-system release of arachidonic acid and other media 48. The immunological features of ferroptosis are damage-associated molecular pattern molecules (DAMPs) before the release of inflammatory mediators 49, such as high-mobility group protein B1.

### Signaling pathway for ferroptosis

Ferroptosis is a ROS-dependent regulatory necrosis, an RCD that relies on iron-mediated oxidative stress and lipid peroxidation-mediated cytotoxic effects. To determine the accumulation of freely active iron and the degree of lipid peroxidation that leads to ferroptosis, multiple transcriptional and posttranscriptional regulatory levels are required. Multiple organelles involved in regulating iron metabolism and redox balance, including mitochondria 50-55, endoplasmic reticulum 7, 56, 57, Golgi 58, and lysosomes 59, 60, indicate a complete signaling network to control and perform ferroptosis. The GPX4 and Xc-system are thought to be the main signaling pathways associated with ferroptosis. The Xc- system transports cystine into cells and synthesizes cysteine (L-cysteine) by a reduction reaction for GSH synthesis. GPX4 is a key enzyme in the antioxidant system, with GSH as the reducing agent, catalyzing peroxide to convert the peroxide bond into a hydroxyl group, converting the peroxide into a lipotoid alcohol, causing it to lose its oxidative activity, thereby inhibiting ferroptosis. Studies have shown that the transcription factors p53 and nuclear factor E2-related factor 2 (NRF2) also play important roles in ferroptosis.

#### p53

p53 can inhibit cystine uptake by downregulating the expression of the Xc-system component SLC7A11, thereby inducing cellular ferroptosis. Nutlin-3, an inhibitor of double-microsynthetic homogen 2, can increase the stability of p53 and maintain intracellular GSH levels through p53-21-dependent pathways, allowing cells to survive metabolic stress, such as cystine loss 61. On the other hand, p53 can inhibit the activity of dipeptidyl peptidase-4 (DPP4), blocking Erastin-induced ferroptosis. Deletion of p53 promotes the interaction of DPP4 with nicotinamide adenine dinucleotide phosphate oxidase 1 (NOX1), which forms the NOX1-DPP4 complex and mediates plasma membrane lipid peroxidation reactions and ferroptosis.

#### NRF2

NRF2 is an important regulator for maintaining intracellular redox homeostasis, upregulating the expression of various genes involved in iron and ROS metabolism (NQO1, HO1, and FTH1) through the p62-Keap1-NRF2 pathway and inhibiting cellular ferroptosis 62. Other studies have shown that SLC7A11 is a transcriptional target of NRF2. Therefore, other genes, such as SLC7A11, are likely to be involved in the NRF2-mediated protective effects of ferroptosis, which requires further research.

#### BECN1

BECN1 is a key factor in autophagy, forming autophagosomes in the early steps of autophagy induction. Studies have shown that BECN1 can regulate cellular ferroptosis. In ferroptosis cells induced by Erastin and sulfasalazine, adenylate-activated protein kinase mediates BECN1 phosphorylation, which binds to xCT in the Xc-system to form the BECN1-xCT complex. This inhibits the activity of the Xc-system, blocking cystine input, and ultimately leading to the occurrence of cellular ferroptosis 40. Different phosphorylation sites will determine whether BECN1 is involved in the BECN1-xCT complex to induce ferroptosis. BECN1-xCT complex-mediated ferroptosis can also be observed in SH-SY5Y neuroblastoma cell lines 41. These studies suggest that BECN1 can promote ferroptosis by inhibiting the activity of the Xc-system.

#### FANCD2

FANCD2, a nuclear protein involved in DNA damage repair, has been found in recent studies to have a regulatory effect on cellular ferroptosis. Song et al. found that ferroptosis occurred in bone marrow stromal cells after FANCD2 deletion 42. On the one hand, they found that the loss of FANCD2 significantly inhibited FTH1 and prostate six transmembrane antigen 3, a metal reductase capable of converting iron from Fe^2+^ to Fe^3+^, which is involved in the metabolism of free Fe^2+^ within cells, such as FTH1. On the other hand, in FANCD2-deficient bone marrow stromal cells, the mRNA expression of GPX4 was slightly downregulated, but its protein expression was significantly suppressed. These two phenomena suggest that FANCD2 can regulate protein expression through transcription-dependent and non-dependent mechanisms, while two different mechanisms ultimately affect the accumulation of Fe^2+^ within cells and the depletion of glutathione. Thus, the FANCD2 gene can regulate ferroptosis by mediating intracellular Fe^2+^ metabolism.

## Neurological Diseases Associated with Ferroptosis

**Figure 1:**
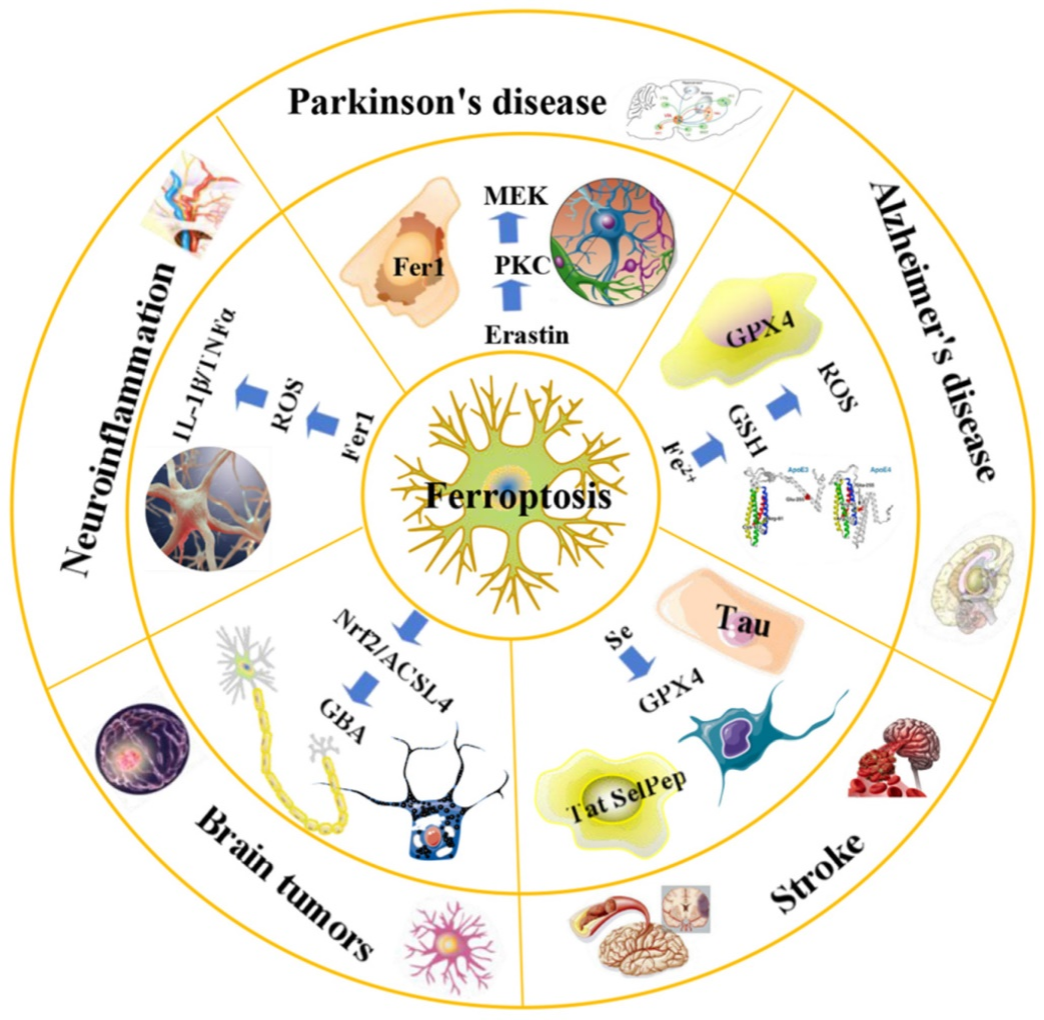
** An overview of ferroptosis and associated neurological diseases.** The treatment of neuroblastoma and meningioma, can be improved by exploiting the effects of ferroptosis. Treatment with Fer-1 can significantly reduce the levels of ROS, IL-1β, TNFα and other inflammatory factors. Ferroportin1 is likely downregulated in the brain tissues of AD patients. The main pathological feature of AD is the deposition of amyloid beta peptide in the brain leading to the death of nerve cells. FTH1 may provide a key link between ferroptosis and ferritinophagy in disease and regulating FTH1 may be therapeutically beneficial in the pathophysiology of PD. Ferroptosis inhibitors can alleviate lipid peroxidation and cell death caused by heme toxicity, and hemorrhagic stroke will show molecular features of ferroptosis.

Ferroptosis is a novel cascade of cell death that was first identified in studies utilizing high-throughput screening to identify antitumor drugs that can kill cancer-causing cell lines through RAS transformation 8, 63. Cells can ingest cystine through the transporter xCT on the membrane surface, which in turn is reduced to cysteine for the synthesis of reduced GSH. Intracellular glutathione peroxidase inhibits ferroptosis by using GSH to remove lipid peroxides that accumulate internally. Thus, inhibiting xCT by small molecule compounds, such as Erastin, or directly removing cystine from the cell culture medium can induce the occurrence of ferroptosis (Figure 1).

Recently, various regulators and regulatory pathways have been discovered, and basic research on ferroptosis has gradually increased, providing new opportunities for the treatment of various diseases 64. Studies have shown that ferroptosis plays an important role in pathological processes, such as tumors, neurodegenerative diseases (e.g., AD/PD) 30, and tissue ischemia-reperfusion injury 15.

### Brain tumors

Cancer has long been considered a genetic disease. However, studies have shown that cancer has the characteristics of many metabolic disorders, exhibiting metabolic abnormalities. Altered iron metabolism is thought to be a marker of cancer 65. Increased intercellular iron input and decreased output are seen in many cancers to support tumor cell survival, rapid cell division, and metastasis 25. Iron reductase plays an important role in the absorption of iron and promotes the development of cancer. Iron has a dual role in cancer, and cancer patients tend to be anemic when they receive chemotherapy, but iron supplementation has the potential to increase tumorigenesis and promote drug resistance. This suggests that iron regulation may improve the prognosis of patients with advanced cancer 66. Studies have shown that iron is associated with various diseases, including cancer. In recent years, the accumulation of iron in chronic inflammatory sites has been considered the root cause of malignant tumors 67.

Glioma is a primary brain tumor produced by glial cells of the central nervous system. Glioblastoma is the most destructive form of brain cancer in humans, and patient survival rates are extremely low. Unfortunately, current treatment options are limited, and gold-standard pharmacotherapy with the chemotherapy drug temozolomide only slightly improves survival. Experimental studies have shown that the efficacy of temozolomide can be improved by inducing ferroptosis. Ferroptosis can also be activated to improve the treatment of the malignant stages of neuroblastoma, meningioma, and glioma 68. Despite their positive effects during chemotherapy, drugs used to induce ferroptosis (e.g., sorafenib) and genetic manipulation of key participants in ferroptosis (e.g., cystine-glutamate exchanger xCT and GPX4) also affect neuronal function and cognitive ability. For patients with glioblastoma, ferroptosis represents an option to improve treatment because these tumors are difficult to cure by radiation, resection, or drug therapy alone, especially due to drug resistance 69. Inducing ferroptosis to limit tumor growth has become a compelling new concept in the treatment of neuroblastoma. In summary, the treatment of advanced cancers, such as neuroblastoma and meningioma, can be improved by exploiting the effects of ferroptosis.

In 2015, scholars conducted experiments on the effect of iron ions on the efficiency of radiotherapy in a mouse model of glioma. Studies have found that iron stimulates the growth of gliomas, and iron chelation reduces blood iron levels by inhibiting ferroptosis, thereby inhibiting iron-stimulated tumor growth 70. Further research confirmed that ferroptosis is a cancer therapy, which mainly induces cancer cell death by promoting the fenton reaction to accelerate the production of ROS 58. There is an increasing number of studies on the mechanism of action between ferroptosis and glioma. In 2019, Gao et al. 71 found that ibuprofen could induce ferroptosis in GBM by downregulating the Nrf2 signaling pathway, thereby inhibiting the viral ability of GBM, that is, triggering ferroptosis became an effective method to eliminate GBM. Recent studies have found that ACSL4 inhibits the increase of glioma cells by activating ferroptosis 72.

### Neuroinflammation

In oxalate-induced mouse models of acute kidney injury (AKI), there is evidence of inflammation in ferroptosis, inhibition of the expression of proinflammatory cytokines (including CXCL-2 and IL-6), and neutrophil infiltration 73. Secondary brain injury (SBI) usually occurs in neurological disorders, such as cerebral hemorrhage (ICH) 74, and its mechanisms mainly include oxidative stress (especially the production of ROS), inflammation, and cell death. Higher amounts of ROS can cause lipid peroxidation and cellular and tissue damage 75, 76. In 2017, researchers discovered that chemical ferroptosis inhibitors, such as Fer-1, deferoxamine, and the vitamin E analog Trolox 77, reduce ICH-induced cytotoxicity when treated *in vitro*. Another study showed that Fer-1 had a significant neuroprotective effect and improved nerve function in collagenase-induced models of cerebral hemorrhage in mice 78. Recently, ferroptosis has been suggested to be related to SBI after cerebral hemorrhage, wherein ferroptosis after cerebral hemorrhage in rats caused an inflammatory response in the brain. Treatment with Fer-1 can significantly reduce the levels of ROS, IL-1β, TNFα and other inflammatory factors, suggesting that inhibiting ferroptosis with Fer-1 can reduce the inflammatory response and improve the neural function of ischemic rats.

GPX4 is a key regulator of ferroptosis, and experimental studies have shown that mice with GPX4 ablation of forebrain neurons have cognitive impairment and neuronal damage and loss at the hippocampal level 5. Through further analysis of the hippocampus, it was found that the lipid peroxidation in the hippocampus was increased, especially the obvious neuroinflammation, which strongly indicated that ferroptosis is the key factor driving hippocampal degeneration 79.

### Alzheimer's disease

Brain cells in patients with AD have been observed to exhibit biochemical and morphological features similar to ferroptosis, including glutathione (GSH) degradation, GPX4 inactivation, increased ROS due to iron metabolism imbalance, lipid peroxidation, and mitochondrial abnormalities 80, and iron metabolism disorders are closely related to A^.^β, SPs, and NFTs. In addition to amyloid plaques and tau protein, dystrophic imbalance of iron is a contributing factor in the pathogenesis of AD 81-83. Disorders of brain iron regulation in AD cause redox-active ferrous to produce hydroxyl radicals in the Fenton reaction and induce/enhance neuroinflammation, possibly leading to oxidative stress and neurodegeneration through ferroptosis 15, 84, 85. These findings suggest that oxidative stress and impaired glutathione antioxidants, with iron homeostasis disorders, play a role in AD and suggest a potential benefit for ferroptosis treatment. Previous studies have proved that Ferroportin1 is likely downregulated in the brain tissues of AD patients 86. Moreover, they reported shrunken mitochondria and other ferroptosis phenotypes in an AD mouse model, and these changes were regulated by pathological Fpn loss in AD 87. Thus, we deduced that elevation of Fpn or amelioration of ferroptosis might be a promising therapeutic approach for AD. Collectively, the latest findings suggest that ferroptosis can provide a successful therapeutic target for alleviating AD 88.

At present, the pathogenesis of AD is still unclear, and the most accepted hypothesis is the amyloid cascade hypothesis, that is, the main pathological feature of AD is the deposition of amyloid beta peptide in the brain leading to the death of nerve cells 89, 90. Deficiency of GSH underlies oxidative stress during aging. In other words, peripheral levels of GSH and protein oxidation are markers of AD progression 91, 92. In 2018, Maher 93 found that Fe^2+^ exacerbates GSH loss while increasing ROS production, and these observations suggest that iron *in vivo* can specifically increase neuronal cell death in the presence of reduced GSH levels. Clinically, magnetic resonance imaging (MRI) was used to detect the elevated iron levels in the hippocampus of AD patients 94, which led to further experimental exploration of the relationship between iron and AD centered on the hippocampus. In conclusion, ferroptosis is one of the main pathogenic mechanisms of AD, and ferroptosis inhibitors have become a research hotspot in the treatment of neurodegenerative diseases such as AD.

### Parkinson's disease

Parkinson's disease is caused by the loss of neurons in multiple areas of the brain, particularly dopaminergic neurons in dense parts of the substantia nigra. Parkinson's disease is mostly treated by restoring dopamine levels in the brain 95. With the continuous deepening of research on the mechanism of PD and ferroptosis, increasing evidence has shown a close connection between PD pathogenesis and ferroptosis mechanisms 96. In patients with PD, elevated iron ion levels may be a cause of disease. Epidemiological studies suggest that both iron intake and environmental iron content are important risk factors for the onset of PD, with a richer iron content being associated with a higher risk of PD 97. In 2017, a study found that the iron content in the nigra part of the brain of PD patients was significantly higher than that of normal people, while the rest of the brain tissue was normal 98. A clinical study of the iron ion chelating agent deferiprone (DFP) showed that iron repellent therapy could alleviate motor symptoms in patients with early PD by reducing iron levels 99. Previous studies have shown that ferritin heavy chain 1 (FTH1) may provide a key link between ferroptosis and ferritinophagy in disease and regulating FTH1 may be therapeutically beneficial in the pathophysiology of PD 100. Another study showed that ferroptosis plays an important role in the early stages of PD and ferroptosis can trigger apoptosis in cell death induced by iron overload 101. In addition, animal studies have shown that iron chelators that can pass through the blood-brain barrier have clear protective effects on PD model mice 102, further demonstrating the correlation between iron content and PD pathogenesis. In summary, the regulatory effect of iron ions on ferroptosis, the correlation between iron content and PD pathogenesis, and the interaction between iron ions and α-synuclein suggest that abnormal iron content may be an important cause of PD.

In 2009, an epidemiological study found that the risk of PD increases in the case of excessive iron intake, suggesting that iron may be one of the risk factors for the development of PD 97. Clinically, susceptibility-weighted imaging (SWI imaging) was used to evaluate iron deposition in the basal ganglia of PD patients, and it was found that iron deposition in the basal ganglia of PD patients would increase with the progression of the disease 103. A new study found that mice treated with MPTP(1-methyl-4-phenyl-1,2,3,6-tetrahydropyridine) Loss of SN dopaminergic neurons and abnormal motor function, accompanied by elevated iron levels in the body and disturbance of peripheral blood iron^104^, further confirmed that iron is closely related to PD. In 2016, Do Van et al. 96 found that ferroptosis is a new form of cell death in PD, which is regulated by protein kinase C (PKC). In other words, the lethality of erastin in dopaminergic cells is due to PKC activation, which promotes ferroptosis as PKC activates MEK signaling. On the other hand, Ferrostatin-1 derivatives and PKC inhibitors can also alleviate the progression of ferroptosis in PD 96, but the mechanism of the related nervous system is not clear at present, further research will be the basis for the development of PD and offers more possibilities for its clinical treatment.

### Stroke

**Figure 2:**
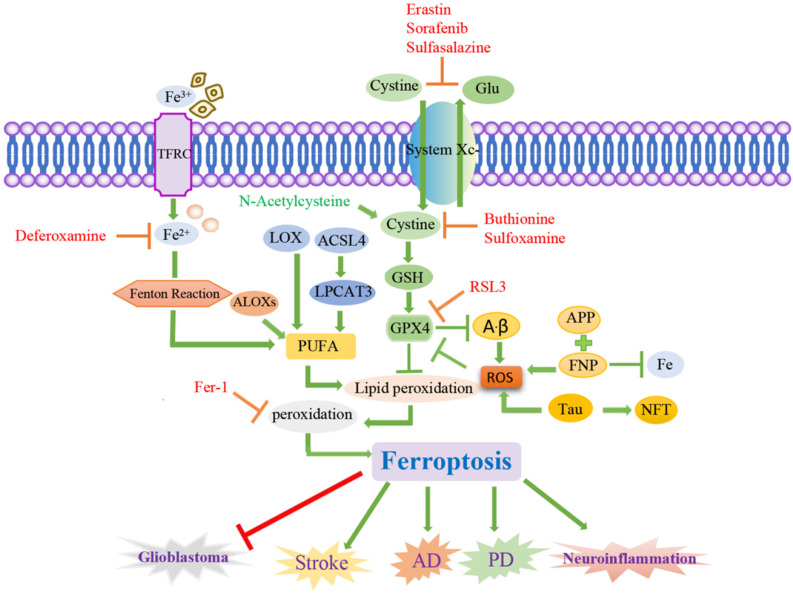
** Signaling pathways of ferroptosis and associated neurological diseases.** Ferroptosis is a cancer therapy, which mainly induces cancer cell death by promoting the fenton reaction to accelerate the production of ROS. ACSL4 inhibits the increase of glioma cells by activating ferroptosis. Chemical ferroptosis inhibitors, such as Fer-1, deferoxamine, and the vitamin E analog Trolox, reduce ICH-induced cytotoxicity when treated *in vitro*. Brain cells in patients with AD have been observed to exhibit biochemical and morphological features similar to ferroptosis, including GSH degradation, GPX4 inactivation, increased ROS due to iron metabolism imbalance, lipid peroxidation, and mitochondrial abnormalities. Ferrostatin-1 derivatives and PKC inhibitors can also alleviate the progression of ferroptosis in PD. Inflammation, excitatory toxicity, iron accumulation and oxidative stress occur during the onset of stroke. The use of iron chelating agents, antioxidants, and free radical scavengers can reduce cerebrovascular damage after stroke.

Stroke is a cerebral vascular disease resulting from insufficient blood supply to the brain, increased cerebrovascular blood pressure or cerebral vascular sclerosis that causes irreversible damage to local brain tissue, accompanied by massive neuronal death. Stroke is the second leading cause of death worldwide and the leading cause of disability worldwide, with a growing incidence 105. At present, studies have confirmed that ferroptosis occurs in neuronal cells during the onset of stroke, ferroptosis mediates the pathophysiological process of stroke, and inhibition of ferroptosis has a protective effect on stroke, which can improve stroke prognosis 106. Clinical studies have shown that inflammation, excitatory toxicity, iron accumulation and oxidative stress occur during the onset of stroke 107. The use of iron chelating agents, antioxidants, and free radical scavengers can reduce cerebrovascular damage after stroke 108, 109. Recent studies have found that hippocampal neurons *in vitro* stroke models and mouse cortical nerve cells after ischemia and reperfusion *in vivo* have the characteristics of ferroptosis, and the use of ferroptosis inhibitors can significantly reverse cell death 77, 110. Ferroptosis plays a key role in stroke, and inhibition of ferroptosis can reverse neuronal damage caused by stroke (Figure 2). A growing number of studies suggest that ferroptosis may be an effective therapeutic target for clinical stroke interventions 111.

The rupture of the blood vessel wall in hemorrhagic stroke leads to the accumulation and lysis of iron-rich red blood cells in the brain parenchyma. The secondary injury model of hemorrhagic stroke was cultured by using the hemoglobin of lysed red blood cells and its oxidation product heme, and this study found that ferroptosis inhibitors can alleviate lipid peroxidation and cell death caused by heme toxicity, and hemorrhagic stroke will show molecular features of ferroptosis 77. On the other hand, ferroptosis inhibitors can also alleviate the nerve cell death caused by iron excess, which indicates that ferroptosis is directly involved in ischemic stroke.

## Drugs Related to Ferroptosis and Their Mechanism of Action

**Figure 3:**
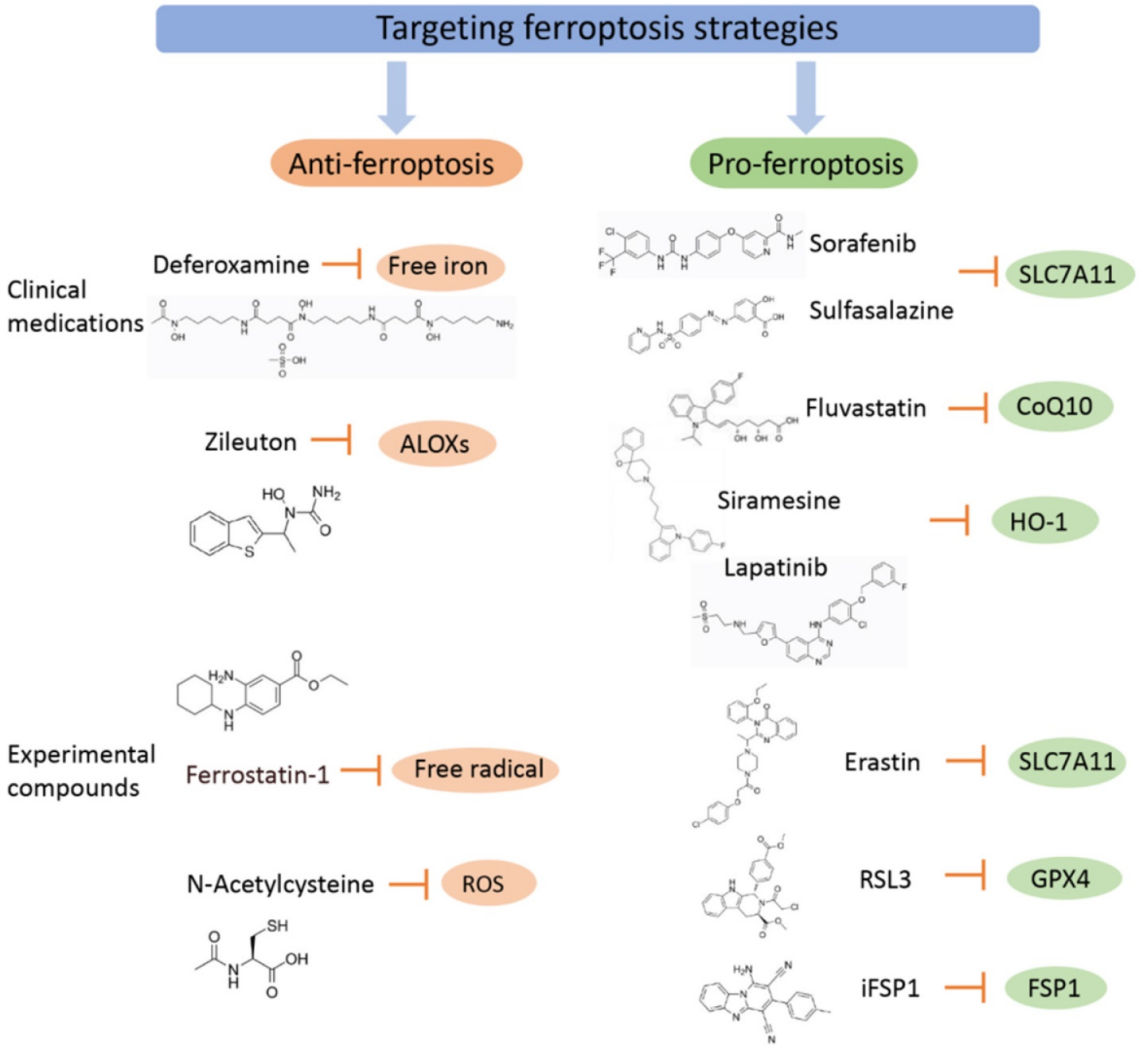
** Targeting ferroptosis strategies (including clinical medications and experimental compounds).** Ferroptosis agonists: The first class includes drugs such as Erastin, sulfasalazine, and sorafenib, among which sorafenib is effectively used in the treatment of liver cancer, and its effect can be inhibited by ferroptosis inhibitors. The second class of drugs directly inhibits or combines GPX4 to induce ferroptosis, and related drugs include the ferroptosis inducers FIN56 and RSL3. The third category includes drugs that reduce intracellular GSH levels, such as cisplatin, which have been used for the treatment of ovarian cancer, lung cancer, thyroid cancer, lymphosarcoma and other cancers. Although other iron chelating agents have also shown blocking effects on ferroptosis *in vitro* experiments or animal models, only DFP has currently entered clinical trials, which may be related to the fact that DFP can cross the blood-brain barrier. Ferroptosis inhibitors exert an anti-inflammatory effect by inhibiting the expression of inflammatory factors in spinal cord contusion (SCI), including IL-1β, TNFα, and ICAM-1.

Since the discovery of ferroptosis, an increasing number of drugs have been shown to exert their efficacy by regulating ferroptosis. Ferroptosis is associated with a variety of physiological pathological processes, especially in the treatment of cancer. Studies have confirmed that ferroptosis plays a key role in killing tumor cells and inhibiting tumor growth. Ferroptosis was identified as the cause of several tumorigenic cell deaths, such as non-small-cell lung cancer 112, breast cancer 113, leukemia 114, pancreatic cancer 115, and hepatocellular carcinoma (Figure 3). Therefore, inducing ferroptosis may be a new cancer treatment strategy.

### Ferroptosis agonists

Sorafenib, sulfasalazine, and artesunate are some of the clinical drugs approved by the U.S. Food and Drug Administration (FDA) that can induce ferroptosis in many cancer types. Clinically, there is an urgent need for drugs that induce new ferroptosis for the treatment of tumors. Inhibitors of ferroptosis reduce the occurrence of ferroptosis in tumor cells and even have a therapeutic effect on some mainstream chemotherapy drugs, such as sorafenib^116^ and cisplatin^117^, greatly reducing the therapeutic effect. Some traditional chemotherapy drugs may inhibit the occurrence of ferroptosis by reducing the Fe^2+^ level in tumor cells, resulting in poor efficacy. Therefore, eliminating inhibitors of ferroptosis and promoting the occurrence of ferroptosis in tumor cells is one of the goals of developing new chemotherapy drugs. At present, three main types of drugs promote the occurrence of ferroptosis by weakening ferroptosis inhibitors: those that directly regulate the level of free iron and weaken the ferroptosis inhibitors present in the iron metabolism process; those that indirectly regulate the intracellular oxidation level by inhibiting molecules in the cell signaling pathway related to antioxidant function; and those that regulate the level of lipid oxidation, reduce the production of intracellular antioxidant substances, and provide a favorable environment for high oxidation levels for ferroptosis.

#### Drugs regulating the level of free iron and their applications

Increased intracellular free iron is a prerequisite for ferroptosis, and reduced transport of iron ions by extracellular transferrin, ferritin binding to free iron, and iron transporters to free iron are the three main ways to reduce intracellular iron ion levels. Lysosomal destroyers (siromecin) and tyrosine kinase inhibitors (lapatinib) can increase the delivery of iron to cells by extracellular transferrin and inhibit the discharge of iron ions dominated by iron transporters, thereby increasing intracellular iron ion levels, promoting ferroptosis in breast cancer cells, and becoming a new drug for the treatment of breast cancer 113. In addition, recent studies have found that cilamethacin and lapatinib can also induce ferroptosis by lowering heme oxygenase-1 (HO-1) levels 118, which indicates that drugs that increase intracellular iron ion levels can also indirectly affect factors related to intracellular oxidation level regulation, thereby enhancing the promotion effect on ferroptosis. Therefore, there is much room for progress in the study of the mechanism of anticancer drugs that regulate iron metabolism.

#### Drugs regulating molecules related to signaling pathways and their applications

Brusatol inhibits the Nrf2 pathway by increasing the ubiquitination and degradation of Nrf2, thereby reducing cysteine levels within cells and promoting ferroptosis 119. Fenugreek (Trigonellne) also has the same effect of inhibiting Nrf2, and it can effectively inhibit cancer cell growth in pancreatic cancer cells *in vitro* 120. Therefore, developing drugs that target molecules that regulate signaling pathways to induce cellular ferroptosis may be an effective cancer treatment strategy.

#### Drugs regulating lipid oxidation and their applications

To date, three broad classes of drugs have been identified that promote ferroptosis by releasing the inhibition of lipid oxidation. The first class of drugs promotes ferroptosis by inhibiting the Xc-system to reduce intracellular cysteine levels and includes drugs such as Erastin, sulfasalazine, and sorafenib, among which sorafenib is effectively used in the treatment of liver cancer, and its effect can be inhibited by ferroptosis inhibitors 118. The second class of drugs directly inhibits or combines GPX4 to induce ferroptosis, and related drugs include the ferroptosis inducers FIN56 and RSL3, although they have not been clinically applied. The third category includes drugs that reduce intracellular GSH levels, such as cisplatin, which have been used for the treatment of ovarian cancer, lung cancer, thyroid cancer, lymphosarcoma and other cancers. Many kinds of drugs regulate lipid oxidation, and their mechanism of action is gradually becoming clear; these drugs are now being applied in the clinical treatment of various tumors.

The above drugs induce ferroptosis by modulating iron homeostasis or by inhibiting the Xc-system-glutathione/GPX4 axis. However, the exact mechanism by which these drugs induce ferroptosis needs further clinical validation.

### Ferroptosis inhibitors

Iron chelating agents can specifically bind free iron ions and promote their excretion, thereby inhibiting the occurrence of ferroptosis and making it possible to reverse the effects of cellular ferroptosis on iron metabolism. Therefore, related drugs that act on iron metabolism pathways and reduce iron content in the body are expected to become new drugs for the treatment of PD. An iron ion chelating agent, DFP, has entered the clinical trial stage 99. Although other iron chelating agents (e.g., DFO, VK-28, M30, etc.) have also shown blocking effects on ferroptosis *in vitro* experiments or animal models 102, only DFP has currently entered clinical trials, which may be related to the fact that DFP can cross the blood-brain barrier. Studies have shown that ferroptosis inhibitors can protect Parkinson's disease mice from peroxidation damage 121. Treatments to prevent ferroptosis have shown therapeutic potential for PD. Prophylaxis for glutathione synthesis rescued neurodegenerative degeneration in mouse models of PD 122, and mild motor improvement was found in early clinical trials of patients with PD 123. Iron chelating agents have also been shown to improve motor symptoms in a variety of PD animal models 20. Ferroptosis inhibitors have shown significant benefits in certain diseases through their anti-inflammatory effects. Ferroptosis inhibitors exert an anti-inflammatory effect by inhibiting the expression of inflammatory factors in spinal cord contusion (SCI), including IL-1β, TNFα, and ICAM-1 124. Ferroptosis inhibitors, such as ferrostatin-1 (Fer-1), are a potential strategy for the treatment of SBI after intracerebral hemorrhage 125. Relevant studies have demonstrated that the anti-inflammatory properties of ferroptosis inhibitors, such as DFP, are beneficial in neurodegenerative diseases, such as Parkinson's disease (PD), motor neuron disease (MND) and Alzheimer's disease (AD) 126, while the role of iron chelation in the treatment of amyotrophic lateral sclerosis (ALS) is currently being explored 127.

Studies have found that the use of iron chelating agents, antioxidants and free radical scavengers can reduce cerebrovascular damage caused by stroke 108, 109. Both hippocampal neurons in an *in vitro* stroke model and mouse cortical nerve cells after ischemia and reperfusion *in vivo* have the characteristics of ferroptosis, and the use of ferroptosis inhibitors can significantly reverse cell death 77, 110.

Recent studies have shown that ferroptosis is of great significance to the occurrence and development of various diseases such as nervous system tumors and neurodegenerative diseases. Inducing or inhibiting ferroptosis through human intervention will provide new ideas for the treatment of these diseases. A variety of compounds have been found to induce or inhibit the occurrence of ferroptosis, known as ferroptosis inducers or inhibitors. Known clinically actionable ferroptosis agonists and antagonists, along with potential beneficial effects, are listed below. First, let's talk about ferroptosis inducers. Erastin is the first ferroptosis-specific inducer discovered, which can directly inhibit the activity of System Xc, affect the synthesis of GSH, and eventually lead to the ferroptosis of human fibrosarcoma cells HT-1080, human foreskin fibroblasts BJeLR, and human lung cancer cells Calu-1 3. Sorafenib is clinically used for the treatment of advanced cancers (such as renal cell carcinoma, hepatocellular carcinoma and thyroid cancer), and it induces ferroptosis in HT-1080 cells in a narrow concentration range 7. Sulfasalazine (SAS) is an FDA-approved first-line anti-inflammatory drug for rheumatoid arthritis. *In vitro*, SAS can specifically inhibit System Xc, significantly inhibit the proliferation of lymphoma cells, indicating that the drug has potential application prospects in the treatment of lymphoblastoma 128. Through bioinformatics analysis, some researchers found that hexamethylmelamine (altretamine), an antitumor drug approved by the US FDA for the treatment of ovarian cancer, can inhibit the lipid repair activity of GPX4, suggesting the potential mechanism of its antitumor activity 129. Platinum compounds such as cisplatin have high affinity for thiol-rich biomolecules and can directly bind to GSH to form Pt-GS complexes, causing GSH depletion and GXP4 inactivation. When cisplatin was used in combination with erastin, human lung cancer cells A549 and human colon cancer cells HCT116 exhibited significant synergistic antitumor activity 112. Recently, studies on osteosarcoma cells have shown that the combined use of other ferroptosis inducers (such as erastin and RSL3) or STAT3 inhibitors can enhance the sensitivity of cells to cisplatin, providing a new idea for the treatment of drug-resistant osteosarcoma 130. The combined use of the lysosome disrupting agent siramesine and the tyrosine kinase inhibitor lapatinib can induce the accumulation of intracellular iron ions by reducing the expression of ferroportin (FPN) and increasing the expression of TF, and induces MDA MB 231, MCF-7, ZR -75 and SKBr3 breast cancer cells undergo ferroptosis, which provides a new strategy for the treatment of breast cancer 113.

Finally, a brief description of ferroptosis inhibitors. XIE et al. 131 found in the experiment of screening ferroptosis inhibitors in the natural product library, that baicalein may inhibit erastin-induced ferroptosis of pancreatic cancer cells by inhibiting GSH depletion, GPX4 degradation and lipid peroxidation. Activates the Nrf2 pathway, prevents erastin-induced Nrf2 degradation, and inhibits oxidative damage. The latest research shows that baicalein can also reduce the oxidation of phosphatidylethanolamine in ferroptosis and improve the prognosis and recovery of cerebral cortical impact 132. Studies have found that rosiglitazone, pioglitazone and troglitazone can specifically inhibit ACSL4 and prevent RSL3-induced ferroptosis and lipid peroxidation in Pfa1 cells. The lipoxygenase inhibitor zileuton is an oral specific inhibitor of 5-LOX for maintenance therapy in patients with asthma. It can protect ACSL4-overexpressing LNCaP and K562 cells from erastin-induced ferroptosis by inhibiting the production of 5-hydroxyeicosatetraenoic acid 133. In addition, the iron chelator deferoxamine (deferoxamine), the cardioprotective drug dexrazoxane (dexrazoxane), etc. also have the effect of inhibiting ferroptosis.

The above are the clinically operable ferroptosis inducers and inhibitors that have been found so far. However, the targets and potential applications of most of these compounds still need to be further understood. Further elucidation of the mechanism of action of these compounds (especially multi-targeted compounds), the relationship between the mechanisms and the characteristics of action, while exploring the possibility of combination therapy and the development of more specific inducers or inhibitors, will have far-reaching impact on its clinical application.

## Opportunities and Challenges

Ferroptosis is an important form of regulated necrosis that is morphologically, biochemically and genetically distinct from other cellular necrosis and apoptosis. The mechanism of ferroptosis is closely related to cellular metabolism, involving a variety of key molecules and signaling pathways. Regulating the synthesis or decomposition of these key molecules and the signaling pathways involved will change the sensitivity of cells to ferroptosis. Reasonable induction or inhibition of cell ferroptosis will help to improve and treat various diseases, especially cancer and nervous system-related diseases. Existing studies have shown that ferroptosis is involved in the process of major systemic diseases. Nervous system diseases mainly lead to the damage and death of nerve cells through the stimulation of neurons, but the mechanism of action of most nervous system diseases is still unclear, and their treatment methods and drug applications are not targeted. Nowadays, more and more experimental studies are based on the role of ferroptosis in neurological diseases, which provides more possibilities for the discovery of potential therapeutic drugs and therapeutic targets for neurological diseases, and also provides further explanation for neurological diseases. Overall, this article reviews ferroptosis and its mechanisms, nervous system diseases associated with ferroptosis, and drugs associated with ferroptosis and their mechanisms of action. However, the research on ferroptosis is still in its initial stage, and there are many problems that have not been elucidated in this paper, and more experiments are needed to deepen its understanding.

Ferroptosis is a special form of cell death that is regulated by multiple genes, affected by multiple signaling pathways, and regulated by intracellular substance metabolism levels and oxidation reactions. Some ferroptosis inhibitors have shown anti-inflammatory activity in experimental models of certain diseases, and the absorption and metabolism of iron have become a target for the treatment of many human diseases, including inflammation and cancer. However, many questions remain. First, what are the key targets and key proteins of ferroptosis? Second, under what circumstances does ferroptosis induce pro-inflammatory or anti-inflammatory effects, as the effect of ferroptosis on the inflammatory response is not absolute? Third, considering that inflammation as a defensive response to injury is not always harmful, is a ferroptosis inhibitor or agonist harmful for anti-inflammatory purposes? Fourth, since ferroptosis is closely linked to cancer and inflammation, does it play a role in inflammation-mediated carcinogenesis? The exact mechanism remains to be confirmed. Fifth, most of the current data come from animal experiments, and it is not yet possible to determine whether the developed drugs are effective or have side effects in the clinic. Sixth, the current research on the intervention effect of ferroptosis on stroke is far from sufficient, and the exact mechanism of ferroptosis after stroke still needs to be studied. Further research should explore how to strictly regulate ferroptosis to more effectively treat cancer and inflammation under related treatment strategies. We believe that further study of the specific mechanism of ferroptosis will be more conducive to the development of new drugs that induce ferroptosis and that in-depth research on ferroptosis will provide a better basis for its clinical application.

## Figures and Tables

**Table 1:** Functions and Classification of Ferroptosis Regulators

Ferroptosis regulators	Function of ferroptosis regulators	Classification of ferroptosis regulators	Refs
Se	Regulates the abundance and activity of GPX4 by synergistically activating the transcription factors TFAP2c and Sp1 to protect neurons	Inhibiting ferroptosis	38
NFE2L2	Regulates the expression of related genes by transactivation to limit oxidative damage during ferroptosis	Inhibiting ferroptosis	39
NADPH	Involved in the circulation of the GSH-GPX4 antioxidant system, and its heavy consumption will limit the antioxidant function of GSH-GPX4	Inducing ferroptosis	40, 41
CoQ10	Can be reduced by ferroptosis suppressor protein 1 (FSP1) to prevent lipid oxidation and inhibit ferroptosis	Inhibiting ferroptosis	42
p53	Inhibit cystine uptake by downregulating expression of the Xc-system component SLC7A11	Inducing ferroptosis	61
NRF2	Upregulate the expression of various genes involved in iron and ROS metabolism (NQO1, HO1, and FTH1) through the p62-Keap1-NRF2 pathway	Inhibiting ferroptosis	62
BECN1	Inhibits the activity of the Xc system, blocking cystine input, and ultimately leading to the occurrence of cellular ferroptosis	Inducing ferroptosis	40, 41
FANCD2	Regulates protein expression through transcription-dependent and non-dependent mechanisms, and ultimately affect the accumulation of Fe2+ within cells and the depletion of glutathione	Inhibiting ferroptosis	42
